# A Central Hospital Board for London

**Published:** 1896-04-18

**Authors:** 


### The Institutional Workshop.

### A CENTRAL HOSPITAL BOARD FOR LONDON.

The question of a Central Hospital Board for London has again been brought forward by the Charity Organisation Society, which held a special meeting to discuss the subject at the Royal United Service Institution on the 13th inst., under the presidency of the Earl of Stamford, the Chairman of the Council of that body. A draft scheme was read to the meeting, but discussion on it was not allowed, the business before the meeting being confined to an expression of opinion on the desirability or otherwise of the establishment of a Central Hospital Board for London. As to what would be the result of this expression of opinion, it was in the course of the discussion stated that a general committee would be appointed by the Charity Organisation Society to go into the details of the scheme, but no definite promise of any further meeting or opportunity for discussion by hospital workers was given. No particulars as to the formation of this committee were offered, except that Mr. Loch (secretary of the Charity Organisation Society) stated that the desire of the society was to have a representative committee, and in reply to a question as to whether a further meeting would be held to appoint the committee, he said that that was a matter for the council of the Charity Organisation Society to decide.

The Chairman (in the absence of Sir James Crichton Browne) moved the following resolution: "That this meeting approves of the establishment of a Central Hospital Board for London on a representative basis."

Mr. Reginald B. Acland seconded, and said that the reasons which had induced him as chairman of the Hospital Saturday Fund to feel the need for a Central Hospital Board were three. First, the immense importance of a better distribution of hospitals; secondly, the want of co-operation amongst hospitals; and, thirdly, the great abuse of the out-patient department. The first two, in his opinion, could best be dealt with by a strong central board, while the third, though it really should be dealt with by the working men themselves, would have, he was afraid, to be taken up from the outside, *i.e.*, by the hospitals themselves, under pressure from a central board.

Mr. R. Brudenell Carter welcomed the main outlines of the proposal laid before the meeting because such a board as was there outlined would work entirely by means of advice and conciliation, and no attempt would be made to coerce or control those who were engaged in the great work of providing medical attendance for the sick.

Mr. Timothy Holmes explained that he was in favour of the principle underlying the resolution before them, though he was not necessarily in favour of the details of the scheme for a central board which had been read. His principal reason in favour of some such scheme was that if the present organisation of voluntary hospitals was allowed to go on much longer, he feared that some such step as municipal control would have to be taken.

Mr. George Brown (as representing the Incorporated Medical Practitioners' Association) also supported the resolution.

The Hon. Sydney Holland (chairman of the Poplar Hospital) was convinced that there was no general feeling of excitement amongst hospital workers in favour of a Central Hospital Board, and he had two reasons for this opinion. First, he had asked a large number of managers and officials their opinion on the subject, to which some had replied that they thought a central board would not do much good, while others had thought that it would not do much harm. The second was the miserably small representation of hospital workers at the meeting that day. He himself thought a Central Hospital Board was needed, chiefly for the reason that there were so many hospital workers doing the same work without being in touch with one another. At the same time it should not be a new board. People were sick to death of boards, as The Hospitals Association and the Charity Organisation themselves showed; and, also, people would not stand boards which existed to advise them how to give their money, and what they ought to do. The proper central board for hospital management, he thought, was the Metropolitan Hospital Sunday Fund Council. Make that council representative of the hospitals, put on it representatives of both the hospitals and the medical profession, and elect from it a proper Distribution Committee, with the power to appoint hospital visitors who will report to the Distribution Committee on whose report the grants to particular hospitals will be based, and they had a solution of the difficulty at present felt.

Objection was also raised by Surgeon-Lieutenant-Colonel Myers on the ground that a board of 160 persons would be quite impracticable, and that though the members of the board might be most enthusiastic at first, that enthusiasm would soon cool off.

Mr. T. Ryan did not think the case for a central board had ever been established, but over and above that any central board should be one appointed not from outside, but by the hospitals themselves. An attempt was to be made shortly to induce the London hospitals themselves to appoint a central authority to advise and counsel them, and such a body was the only body which would now be practicable.

In the result the resolution was declared carried.

